# Ethics assessment in research proposals adopting CRISPR technology

**DOI:** 10.11613/BM.2019.020202

**Published:** 2019-06-15

**Authors:** Francois Hirsch, Ron Iphofen, Zvonimir Koporc

**Affiliations:** 1 Ethics Committee of the French National Institute of Health and Medical Research (INSERM), Paris, France; 2 Independent Research Consultant, La Rochelle, France; 3 Catholic University of Croatia, Zagreb, Croatia

**Keywords:** CRISPR/Cas9, genome editing, ethical review

## Abstract

The rapid and exponential growth of genome editing has posed many challenges for bioethics. This article briefly explains the nature of the technique and the particularly rapid development of Clustered Regularly Interspaced Short Palindromic Repeat (CRISPR) technology. The international and, specifically, European-level systems for assessing the ethical issues consequent on these developments are outlined and discussed. The challenges posed by cases in China are summarized to raise concerns about how a more shared, universally consistent appraisal of bioethical issues can be promoted.

## What is the technology at stake?

Genome editing is amongst many technologies aimed at better understanding the genome and developing better techniques to make changes in the genome. However, only a few scientists are currently engaged in human gene editing since efficient tools for therapeutic purposes have only recently become available. Zinc finger proteins (ZNFs) were the first of the “genome editing” nucleases described to control gene expression in mammalian cells, then rapidly scientists proposed a simpler approach based on the use of transcription activator-like effector nucleases (TALENs) (*1*, *2*). Despite several clinical trials conducted based on these technologies, the development of both techniques was limited by their cost and their technical drawbacks (*3*). In parallel, in 1993, Frederico Mojica of the University of Alicante (Spain) described a curious phenomenon set highlighted by a few scientists, allowing bacteria to expel the virus infecting them by creating a true memory of these attacks, and named this mechanism ‘CRISPR’ (Clustered Regularly Interspaced Short Palindromic Repeat) (*4*). When a bacterium is attacked by a virus it protects itself by cutting the viral DNA and memorizes it by storing a few fragments. Clustered Regularly Interspaced Short Palindromic Repeat thus resembles a hard drive, which stores these fragments. If the bacterium is attacked again by the same virus, it has its ‘identity card’ and ‘memories’ fragments which will act like a magnet by recognizing the DNA of the virus and allow the recruitment of molecular scissors, the enzyme Cas, which cuts and destroys the virus. The bacterium is thus protected from the virus.

Clustered Regularly Interspaced Short Palindromic Repeat research quietly continued up to 2012 when several teams customized the bacterial system to use it for the accurate, cheap and quick target modification of the genome of eukaryotic cells (*5*). In relatively few years, this technique has been applied to human, animal, fungal and plant cells, leading to multiple applications (*6*-*10*).

## CRISPR in humans

In humans, an article published in August 2015 by a Chinese team, demonstrating the ability to manipulate human embryos *in vitro* using CRISPR, was the first reported study to raise some serious ethical and social concerns on the potential consequences of the technology (*11*). These researches attempted to change the β-thalassemia gene in non-viable human embryos obtained in clinics; however, only a few of the manipulated embryos expressed the gene. Despite the limitations of the approach chosen by these researchers, this study demonstrated that human embryonic cells can be changed in a simple way, and an article published by an American team in collaboration with Korean scientists followed, reinforcing the idea that human germ cells could be changed by the CRISPR technology (*12*). These studies also presented for the first time an *ad hoc* creation of human embryos for research purposes, an illegal procedure in most EU countries adhering to the Convention for the Protection of Human Rights and Dignity of the Human Being with regard to the Application of Biology and Medicine: Convention on Human Rights and Biomedicine, signed in Oviedo in 1997 (*13*). Of note, the United States’ National Academy of Medicine and the National Academy of Sciences have both given the ‘go ahead’ for genetically altering human embryos as long as numerous conditions are fulfilled, including when no other treatments are available for the disorders they carried, and when sufficient safety guarantees are met (*14*). More recently, in August 2018, researchers from Weill Cornell University reported at the annual meeting of the European Society of Human Reproduction and Embryology (ESHRE), that DNA in sperm could be fixed with CRISPR using a brief but powerful electrical shock, without killing sperm cells; the corresponding publication is awaited. The main ethical question raised here is the heritable nature of any gamete modification, an intervention prohibited by Article 13 of the Oviedo Convention.

Things considerably evolved on November 28th, when at the Second International Summit on Human Genome Editing in Hong Kong, a Chinese scientist, Dr. He Jiankui, announced the birth of twins (girls Lulu and Nana) from embryos whose genome had been modified prior to their transfer into the womb. The modification was done using CRISPR-Cas9 disabling copies of the CCR_5_ gene in human embryos with the stated aim of preventing the transmission of a HIV infection from the father’s side.

While on the face of it this seems a reasonable moral justification for the action taken, the lack of any confirmed assessment of the implications of the action casts doubt on the scientist’s motives and has further raised international concerns about the premature application of such a ground-breaking technology. Even though Dr. He did not initially present any solid proof or witnessed confirmation that this experiment on human embryos had really taken place, it was unsurprising that a cascade of interest in the media and reactions from different institutions, research ethics boards, scientists and professionals all over the world was triggered. It appeared as though the world suddenly awoke to the realization of the sharp edge between promising science breakthroughs and a murky abyss of risky experiments, which the future of humankind could easily be pulled into. The lack of ethical procedures, assessments and guidance, which characterized this project, shocked the world scientific community. To change the public perception on his actions, Dr. He tried to justify his work in a series of video interviews available online (*15*). One of his arguments was that the procedure was safe and that no other genes were affected. However, targeting the CCR5 gene is just one potential route of virus cell entry clearly pointing out that this procedure could not be seen solely as a consequence of a real medical need, rather more like a *proof of concept* (*16*). More precisely this was an experiment on human embryos that could not be seen as absolutely necessary for the care of the unborn.

## Methods

In order to assess the actions and commentary in this field we conducted searches through PubMed to retrieve a total number of publications containing word CRISPR in their abstracts or in their keywords during the period from 2002 up to 2018 (the search being conducted at the end of February 2019). For the purpose of a focus on the EU-model, data on proposals/projects having CRISPR in the keywords or mentioned in abstracts were received by the courtesy of the Ethics and Research Integrity Sector, Directorate General for Research & Innovation of the European Commission (EC) from the EC statistic tool called ‘Corda’ (as of the end of January 2019). Data for 2018 are incomplete and thus not fully visible in Corda since the negotiating phase for some proposals applied for in 2018 was still ongoing at the time of this paper submission. We here maintain the difference between terms ‘proposal’ and ‘project’. Only those applications, which, at the end of the evaluation process, are chosen to sign the grant agreement and will be funded, are considered as “projects”. All others we refer to as “proposals”.

## How to address these ethical challenges

### Through an international debate

The perception of the nature and value of human embryos differs between societies, cultures and religions. The isolation of human embryonic stem cells (hESCs) from the human embryo is considered highly objectionable and contested as it requires the destruction of the human embryo leading to the banning of hESC research in many countries around the world (*17*, *18*). The national laws on the use of hESC are not globally standardized (*19*). Even at the EU level, laws on hESC research differ substantially from the very strict in, say, Germany, Austria and Croatia to the more supportive of research in Greece, Sweden and the UK (*20*). In some countries, there is ongoing debate about whether the current legislation should be amended. For example, recently the US National Institutes of Health (NIH) announced its plans to lift its moratorium on funding research that involves injecting human embryonic stem cells into animal embryos, which would allow for the creation of part-human and part-animal organisms known as chimeras (*21*).

The International ethical guidelines for health-related research involving humans are well established (prepared by the Council for International Organizations of Medical Sciences (CIOMS) in collaboration with the World Health Organization (WHO)) (*22*). The ‘China Twins Case’ clearly demonstrated not only a lack of appropriate implementation of this guideline, but rather a more complete avoidance of any relevant ethics procedure for work with humans and hESC since this would have clearly led to the banning of such experiments. The potential unknown irremediable risks to foetuses and future generations of the CRISPR/Cas9 technique used for genetic modification in human embryos requires a general consensus and adequate regulations before human germline genome editing is introduced worldwide (*23*). Importantly, in July of 2017, an extensive report on the social and ethical issues raised using genome editing as a technology that could influence inherited characteristics in humans was published by the Nuffield Council on Bioethics in the UK (*24*). Unfortunately, Dr. He together with his research team fully disregarded all warnings coming from that report, initiating a profoundly challenging experiment and irretrievably changing the public perception of CRISPR/Cas 9 technique while potentially damaging future positive and constructive work in this field.

Worldwide academic, professional and public reaction was immense. Almost simultaneously many eminent world public and governmental organizations together with different scientific and academic institutions released their official announcements (*25*). The official statement of the Organizing Committee of the Second International Summit on Human Genome Editing recommended an independent assessment to verify Dr. Jiankui He’s claims and to ascertain whether the claimed DNA modifications had actually occurred. The Organizing Committee clearly stated that “…even if the modifications are verified, the procedure was irresponsible and failed to conform with international norms. Its flaws include an inadequate medical indication, a poorly designed study protocol, a failure to meet ethical standards for protecting the welfare of research subjects, and a lack of transparency in the development, review, and conduct of the clinical procedures.” (*26*). There was no explanation of Dr. He’s proceedings even from the Chinese official authorities. Both the Genetics Society of China and the Chinese Society for Stem Cell Research jointly issued a statement saying that the experiment “violates internationally accepted ethical principles regulating human experimentation and human rights law” (*27*). Finally, in January 2019, it was officially confirmed that Dr. He’s experiments had actually taken place. Authorities and investigators confirmed that the twin girls were born and were under medical observation. Moreover, an announced additional pregnancy was also confirmed and was being monitored – meaning that another new gene-edited baby is to be arriving soon (*28*). Chinese authorities are still investigating He and the responsibility of other researchers who had knowledge of his actions became hotly debated. In light of these events, it is questionable how public scrutiny will affect the future of work in the field (*29*). For our purposes there are obviously major concerns about the potential irreversible modification of the human genome and the possibility of destroying several animal species deemed ‘un-necessary’ while such experimentation progresses. In 2015, representatives of the U.S. National Academy of Sciences and Medicine, the Chinese Academy of Sciences and the UK Royal Society met in Washington DC. They released a statement pointing to the critical social issues raised by these technologies but did not call for a moratorium as it was deemed unrealistic (*30*). However, during the 13th Conference of the Parties to the Convention on Biological Diversity in Cancún, Mexico in December 2016, more than 170 non-governmental organizations called for a moratorium on the modification of animals by the gene drive approach, as long as the environmental impacts were not more fully and precisely considered (*31*).

In Europe, many academies and institutions were also concerned by this possible irreversible modification of the human genome in contravention of Article 13 of the Oviedo Convention. The INSERM Ethics Committee (IEC), in France, also initiated a reflection at the national and international level, involving experts living in economically vulnerable countries that are or could be impacted by these technologies. The group’s initial position was published in the journal *Nature* in early 2017 and a more elaborated one in *Transgenic Research* was released in July 2017 (*32*, *33*). Among its recommendations, the group called for the creation of an international association which has since been launched on March 23, 2018, in Paris: ARRIGE (Association for Responsible Research and Innovation in Genome Editing) (*34*). This association has a mission to inform the general public and policymakers on the real issues of the development of genome editing techniques, by creating several thematic focus groups. This reflection is to focus on researching the methods of assessment of the effectiveness and safety of techniques, on the assessment of the impacts of the use of the ‘gene drive’ on the acculturation of the scientific community in the development of a responsible innovation, and on a transparent and honest communication to the general public. The ARRIGE initiative links with other similar initiatives that were launched in the USA, and several papers have reported on its activities (*35*-*38*).

### By a thorough ethics review of the projects: the EU model

**Figure 1:**
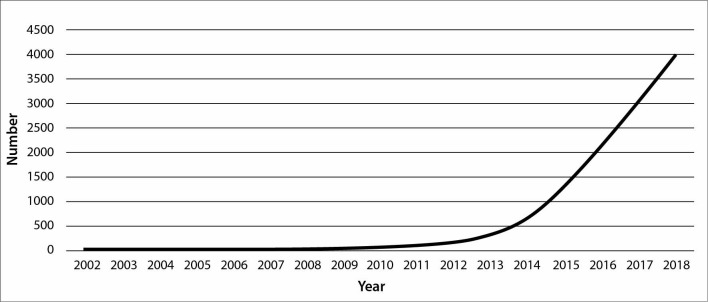
Number of published papers in PubMed containing word CRISPR in title or in abstract *per* year. CRISPR - Clustered Regularly Interspaced Short Palindromic Repeat.

Without inferring any malicious intent on the part of Dr He, it is clear that there is consensus across the international community to find a way to prevent any similar ill-considered experiments in the future until the corresponding safety, scientific, ethical and legal aspects have been discussed openly and broadly and their implications fully understood. That task will not be easy since the number of research projects involving the CRISPR technology has grown rapidly over the last few years. Data retrieved from PubMed at the end of February 2018 shows a significant growth in the number of published papers during the period from 2002 up to 2018 containing the word CRISPR (Figure 1).

**Figure 2:**
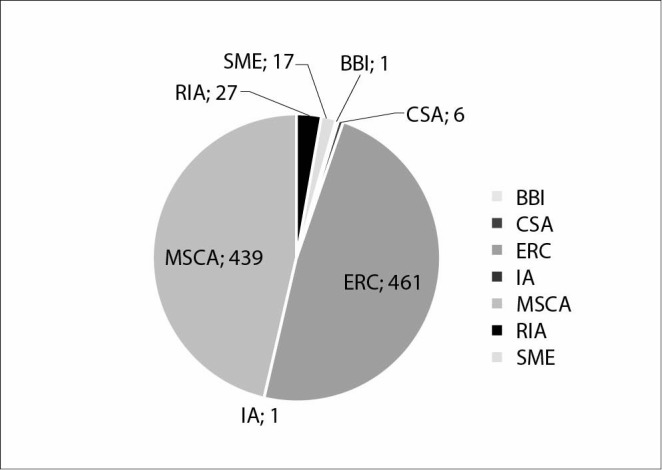
Number of H2020 CRISPR proposals *per* H2020 action. CRISPR - Clustered Regularly Interspaced Short Palindromic Repeat. BBI - Bio-Based Industries. CSA - Coordination and Support Action. ERC - European Research Council. IA - Innovation Action. MSCA - Marie Sklodowska-Curie actions. RIA - Research and Innovation Actions. SME - Small and Medium-sized enterprises.

Data supplied to the authors by the courtesy of Ethics and Research Integrity Sector/DG Research & Innovation of the European Commission from the EC statistic tool called Corda showed 952 proposals and projects with CRISPR in the title, keywords or mentioned in the abstracts (data collection performed at the beginning of February 2019). Their distribution under specific H2020 Framework Programme actions: Bio-Based Industries (BBI), Coordination and Support Action (CSA), Innovation Action (IA), Marie Sklodowska-Curie actions (MSCA), Research and Innovation Actions (RIA), Small and Medium-sized Enterprises (SME) and the European Research Council (ERC) is shown in Figure 2. These numbers clearly demonstrate that the wide use of CRISPR technology has already brought the world in to the new ‘CRISPR era’ aiming to help to prolong the human lifespan in general. However, the question remains as to whether society in general is also prepared for the unanticipated consequences of the potential unethical application of such prominent and pervasive technology. An appropriate and effective ethics review process is essential to help to conceive of all possible ethics issues arising out of the use of CRISPR technology. Such an ethics review process must be efficient, not necessarily blocking the innovative project cycle but still having enough strength to stop it at any point in time in case any violation of the agreed ethics norms and principles is detected.

The Ethics Assessment process established by the European Commission (EC) and conducted on all projects applying for funding by the European Commission, Horizon 2020 (H2020) and European Research Council (ERC), is carefully tailored to detect and prevent any possible destruction or experimental creation of new human embryos. At the same time, that assessment procedure leaves enough space to allow the use of CRISPR technology for scientific research purposes under the condition that all possible ethics issues arising in such projects are appropriately dealt with. For instance, an initial ethics screening by two independent experts is implemented in the very early phase of project applications. Projects which will be funded, and which are identified as “ethically sensitive”, must then go through a more detailed Ethics Review procedure guided by more external independent ethics experts. Only the projects which are thoroughly scanned by an independent and multidisciplinary Ethics Review Panel, consisting of 5-6 independent ethics experts, and which completely fulfil all ethics requirements, can receive the ‘ethics clearance’ for preparation of the Grant Agreement. The ethics monitoring process does not stop at that time point. A procedure of ‘Ethics Check/Ethics Audit’ enables the European Commission to closely monitor project implementation, having the possibility to completely stop the project process at any further time point in case the ethics dimensions of the project are not respected. As shown in Table 1 and Table 2 all funded and previously ethically screened CRISPR projects eventually went through the full Ethics Panel Review (data presented up to 2017), which is sometimes due to the grant agreement negotiation phase, and thus postponed to the next calendar year.

**Table 1:** Number of CRISPR projects/proposals *per* H2020 action

**Category**	**Year**					**Total (N)**
	**2014**	**2015**	**2016**	**2017**	**2018** **(incomplete data)**	
**Funded projects (N)**	32	44	57	54	9	196
**Proposals not funded due to limited foundation resources (N)**	40	96	160	103	13	412
**Rejected proposals (N)**	35	69	54	117	37	312
**Proposals on reserve list (N)**	5	12	7	7	1	32
**Total (N)**	112	221	278	281	60	952
CRISPR - Clustered Regularly Interspaced Short Palindromic Repeat.						

**Table 2:** Total number of H2020 CRISPR projects (signed Grant Agreement) which went thru the Ethics Review in period from 2014-2017

**Year**	**Ethics reviewed CRISPR projects (N)**
2014	31
2015	49
2016	56
2017	52
Total (N)	188
CRISPR - Clustered Regularly Interspaced Short Palindromic Repeat.	

This data clearly shows a serious and thorough approach of the EC Ethics Review procedures, conducted on projects carrying the CRISPR technology. The growth in H2020 applications for proposals carrying the CRISPR technology is undoubtedly likely to continue to increase. Ethics review must be applied to novel developments in this field to prevent possible violations of ethics principles and to ensure that review process will address the inevitable emergent ethical concerns as the technology grows and develops. All too often ethical concerns ‘follow’ innovation since it remains impossible to anticipate all the problems arising, especially from novel biotechnologies. As with all technological innovation, biotechnology must face the ethical hurdle of the Collingridge dilemma: impacts cannot be easily predicted until the technology is extensively developed and widely used, but controlling or changing the method is difficult when the technology has become entrenched (*39*). The spirit of ethics review is not to block the testing and implementation of new technologies but to ensure the effective monitoring of projects and to facilitate scientific progress that remains within accepted moral boundaries. Of course, all ethics review procedures could be improved, and we cannot assume that those applied by the European Commission are necessarily the ‘best’. However, we are assured that they themselves remain under constant review and must adapt to new and emergent technologies in all fields. The He’s case illustrates what can happen when the spirit and practice of supportive review is ignored or sidestepped. More importantly formal ethics review systems will never be adequate to control researchers whose passion to move quickly overcomes their willingness to ensure that the measured, balanced and constructive support that is possible from an ethical review process that engenders a facilitative culture is provided. That must come from a culture of professional scientific integrity that ultimately recognizes that ethical research lies in the hands of the researcher in the field and in the laboratory - ethical review can only ever act as a further, mutually supportive balance on the eagerness of the scientist whose passion must be held in check to ensure that both societal and individual benefit is secured. Finally, we can conclude that the crucial element for responsible and ethical use of CRISPR Genome Editing tools lies with the researchers themselves. Researcher ethics awareness is essential so that good fruits from the tree of science can be obtained.
